# The effectiveness of computerized clinical guidelines in the process of care: a systematic review

**DOI:** 10.1186/1472-6963-10-2

**Published:** 2010-01-04

**Authors:** Gianfranco Damiani, Luigi Pinnarelli, Simona C Colosimo, Roberta Almiento, Lorella Sicuro, Rocco Galasso, Lorenzo Sommella, Walter Ricciardi

**Affiliations:** 1 Department of Public Health-Università Cattolica Sacro Cuore-Rome, Largo Francesco Vito 1, 00168, Rome, Italy; 2 San Filippo Neri-Hospital Trust-Rome, Italy, Piazza di Santa Maria della Pietà 5, 00135, Rome, Italy; 3 Oncological Referral Center of Basilicata (IRCCS CROB), Via Padre Pio 1, 85028, Rionero in Vulture, Potenza, Italy

## Abstract

**Background:**

Clinical practice guidelines have been developed aiming to improve the quality of care. The implementation of the computerized clinical guidelines (CCG) has been supported by the development of computerized clinical decision support systems.

This systematic review assesses the impact of CCG on the process of care compared with non-computerized clinical guidelines.

**Methods:**

Specific features of CCG were studied through an extensive search of scientific literature, querying electronic databases: Pubmed/Medline, Embase and Cochrane Controlled Trials Register. A multivariable logistic regression was carried out to evaluate the association of CCG's features with positive effect on the process of care.

**Results:**

Forty-five articles were selected. The logistic model showed that Automatic provision of recommendation in electronic version as part of clinician workflow (Odds Ratio [OR]= 17.5; 95% confidence interval [CI]: 1.6-193.7) and Publication Year (OR = 6.7; 95%CI: 1.3-34.3) were statistically significant predictors.

**Conclusions:**

From the research that has been carried out, we can conclude that after implementation of CCG significant improvements in process of care are shown. Our findings also suggest clinicians, managers and other health care decision makers which features of CCG might improve the structure of computerized system.

## Background

Clinical practice guidelines have been developed to improve the quality of care, patient access, treatment outcomes, appropriateness of care and achieve cost containment by improving the cost benefit ratio [1-4].

At the same time many healthcare organizations have widely promoted the development of computerized clinical decision support systems (CDSS) with the aim of improving practitioners' performance [5-7].

According to the indications of regulatory systems, professional bodies and consumer organizations, CDSS can also support the implementation of the computerized clinical guidelines (CCG) [8].

An effective model of CCG consists of computer accessibility, patient-specific reminders in the clinician's workflow and its integration with medical records, as demonstrated by Wang et al. [9].

Even though different studies in literature are focused on demonstrating that CDSS can have an impact on physicians' behaviour regarding to patients' care [6,10-12], there are very little evidence about the effectiveness of electronic guidelines [13-15] and impact of computerized support on implementing of clinical recommendations. In a qualitative systematic review, Shiffman et al. [13] highlighted higher effect of CCG versus non-electronic systems. Due to the lack of studies containing quantitative evaluation, research was focused on a systematic review of available literature about the impact of CCG upon the process of care compared with non-computerized clinical guidelines (NCCG) (such as paper guidelines, peer-to-peer consultation and previous experience.). Moreover, were analysed specific features of the computerized guidelines which are potentially linked with the improvement of the process of care.

## Methods

### Search strategy

An extensive search of scientific literature was carried out querying electronic databases to identify relevant studies: Pubmed/Medline, Embase and Cochrane Controlled Trials Register. The search covered the period from January 1992 to March 2006. The search of articles were carried out using the following key words, related to:

1. Exposure variables: computerized clinical guidelines, computer-based guidelines, computerized clinical recommendations, computer decision support aids, software guidelines, computerized clinical pathways, computerized critical pathways, computer-based pathway*, electronic care map, electronic care pathways, electronic clinical pathways, electronic critical pathways, integrated care pathway, electronic clinical reminder, electronic clinical reminders, electronic reminder AND practice guidelines AND electronic medical record, computerized reminders AND guidelines;

2. Effect variables: medical outcomes, organisation's outcomes, patients' outcomes; process of care

3. Population variables: medical doctors, health personnel.

The search in grey literature was carried out using general purpose search engines (GOOGLE, VIVISIMO) in order to identify missing articles. Rest of the articles were identified through the analysis of bibliographic citations.

### Inclusion and exclusion criteria

Considering study's design, only experimental or analytical studies were included while descriptive studies were excluded. The main exposure variable in our research was the comparison between CCG and NCCG (such as paper guidelines, peer-to-peer consultation and previous experience). Papers which did not contain comparison between CCG and NCCG were excluded from analysis. So, only the papers in which the guidelines were coming from a scientific society recommendations or approved by a National body, a scientific society, union or corporation of physicians or universities were included in this study. Articles not matching these criteria were excluded. Also, only articles focusing on adult patients (age ≥18 years) were taken into consideration. Studies involving children and adolescents (age <18 years) were excluded due to the fact that there are specific factors linked with the paediatricians adherence to guidelines in this specific age group [16-18].

### Study selection

Titles and abstracts of the selected studies were reviewed independently by two authors (C.S.C and A.R.) and were rated as "potentially relevant" or "not relevant" using search strategies based on study design, subjects and type of intervention. If one of the reviewers considered a reference potentially relevant, full-text articles were retrieved and examined independently, using the full set of inclusion and exclusion criteria to select the final number of studies for research. Disagreements between reviewers were resolved by discussion or by third author (G.D.).

### Data extraction

Two reviewers (C.S.C. and A.R.) assessed whether the use of computerized guidelines was going to improve the process of care and evaluated the positive or negative impact of computerized guidelines on the process of care.

Afterwards, the outcomes were distributed in two groups: favouring CCG and favouring NCCG based on evaluation of results of inference analysis. Then, the effect of computerized guidelines was defined as positive when reported improvement was more than 50% of the outcomes. Effect was defined as negative when the improvement was equal or less than 50% of the outcomes. Positive or negative effects of computerized guidelines were confirmed according to the authors' judgment in the conclusions' section of each single paper. Finally, were analysed the variables potentially linked to positive effect on the process of care. Some variables were not included in the analysis because it was not possible to obtain them from most studies (patients' age, health care givers' age, health care providers' degree, duration of observation). The analysed system's features were identified referring to Kawamoto [12] or they were extracted by the authors from the studies. So, 21 features related to the following categories were analysed:

• General system features;

• Clinician-system interaction features;

• Communication content features;

• Auxiliary features;

• Guidelines features.

The description of each feature is reported in Table 1.

**Table 1:** Description of 21 CCG's features in five categories and proportion^# ^of study containing each feature

CATEGORY	FEATURE	EXPLANATION	PROPORTION
**General system features**	Presence of networks *	User has access to recommendation in computer terminals, available at several workstations in the hospital.	0.20
	
	Type of suggestion *	Recommendation is provided in different ways including reminders of overdue health care tasks, alerts of critical values, prompts for various active care issues.	0.78
	
	Conflict of interest *	Software designer or producer is involved in the design of study.	0.38
	
	Degree of automation *	User automatically receives prompts (complete automation) instead of active initiation of the system by user (incomplete automation).	0.80

**Clinician-system interaction feature**	Automatic provision of recommendation in paper version as part of clinician workflow **	Recommendations printed on paper forms and attached to patient charts by clinical support staff, so that clinicians do not need to look for the computer advice.	0.29
	
	Automatic provision of recommendation in electronic version as part of clinician workflow **	Electronic recommendations linked to patient charts display automatically to clinicians when a clinician accesses the database.	0.82
	
	Data updating via network *	Data of patient are updated via network link to servers storing information about all contacts of patient with the hospital.	0.33
	
	Request documentation of the reason for not following recommendation **	The user is asked to justify the decision of disagreement with a reason such as "the patient refused" or "I disagree with the recommendation".	0.56
	
	Provision of recommendation at time and location of decision making **	Recommendations provided as chart reminders during an encounter, rather than as monthly reports listing all the patients in need of services.	0.13
	
	Recommendation executed by noting agreement **	Computerised system provides recommendations in response to an order and the user simply clicks "OK" to order the recommended tests.	0.11

**Communication content features**	Provision of a recommendation, not just an assessment **	Systems show better actions to perform, rather than simply providing a diagnosis.	0.11
	
	Promotion of action rather than inaction **	Systems recommend an alternative view, rather than simply recommending the order to be cancelled.	0.11
	
	Justification of recommendation via provision of reasoning **	Recommendation for a check justified by noting date of last exam and recommended frequency of testing.	0.18

**Auxiliary features**	Local user involvement in development process **	Recommendation design finalised after testing preliminary versions of software (beta version) with representatives from targeted user group.	0.09
	
	Provision of recommendations to patients as well as providers **	As well as providing chart reminders for clinicians, system generates postcards that are sent to patients to inform them of existing recommendation.	0.18
	
	Recommendation accompanied by periodic performance feedback **	Users are sent e-mails periodically that summarise users compliance with recommendations.	0.02
	
	Recommendation accompanied by conventional education **	Implementation of a recommendation is accompanied by a presentation or an appropriate explanation for following such suggestion.	0.27
	
	User training *	A training period is provided for users to experience the basic features of the software.	0.22

**Guidelines features**	Type of guideline*	Recommendations are focused on preventive or treatment issues or both options.	0.31 0.62 0.07
	
	Type of condition*	Recommendations are oriented towards acute or chronic patients or both options.	0.16 0.60 0.24
	
	Type of intervention*	Recommendations suggest to administrate tests or/and drugs to patients or to perform other type of intervention on them or both options.	0.53 0.16 0.31

** Feature referring to Kawamoto

* Feature selected by authors

# Calculated on the total of 45 studies

### Quality assessment

The methodology of each study was assessed independently by two authors (C.S. and A.R.) according to a score assessing five potential sources of study bias [10,19-21]. Disagreements were solved by consulting the third author (G.D.) or according to a consensus. The studies were evaluated using following system:

- allocation to study groups (random, 2; quasi-random, 1; selected concurrent controls, 0);

- data analysis and presentation of results (appropriate statistical analysis and clear presentation of results, 2; inappropriate statistical analysis or unclear presentation of results, 1; inappropriate statistical analysis and unclear presentation of results, 0);

- presence of baseline differences between the groups that were potentially linked to study outcomes (no baseline differences present or appropriate statistical adjustments made for differences, 2; baseline differences present and no statistical adjustments made, 1; baseline characteristics not reported, 0);

- objectivity of the outcome (objective outcomes or subjective outcomes with blinded assessment, 2; subjective outcomes with no blinding but clearly defined assessment criteria, 1; subjective outcomes with no blinding and poorly defined, 0);

- completeness of follow-up for the appropriate unit of analysis (> 90%, 2; from 80% to 90%, 1; < 80% or not described, 0).

The cut-off value for including an article in our paper was 5/10.

The quality assessment of each study is reported in Table 2.

**Table 2:** Study design and quality assessment of selected articles

Quality assessment									
**Authors**	**Year of publication**	**Rivista**	**Study Design**	**Method of allocation to study group**	**Data analysis and results**	**Presence of baseline differences between groups potentially linked to study outcome**	**Type of outcome measure**	**Completeness of follow-up**	**Total**

Burack	1997	Medical Care	Experimental	2	2	2	2	2	**10**

Burack	1994	Medical Care	Experimental	2	2	2	2	2	**10**

Butzlaff	2003	Family Practice	Experimental	2	1	2	0	2	**7**

Cannon	2000	JAMIA	Experimental	2	2	2	1	2	**9**

Carton	2002	Clinical Radiology	Observational (time series)	0	1	0	2	2	**5**

Dayton	2000	Medical Decision Making	Experimental	2	1	0	0	2	**5**

Demakis	2000	JAMA	Experimental	2	2	2	2	2	**10**

Derose	2005	American Journal Manag Care	Experimental	2	1	0	2	2	**7**

Dexter	2001	New England Journal of Medicine	Experimental	2	2	2	2	2	**10**

Durieux	2000	JAMA	Observational (time series)	0	1	1	2	2	**6**

Feldman	2005	Health Services Research	Experimental	1	1	2	1	2	**7**

Feldstein	2006	Journal American Geriatric Soc	Experimental	2	1	2	2	2	**9**

Filippi	2003	Diabetes Care	Experimental	2	1	0	2	2	**7**

Fitzamaurice	2000	Arch Intern Med	Experimental	2	1	2	1	2	**8**

Frank	2004	Australia	Experimental	1	1	2	2	2	**8**

Hetlevik	1999	Scand J Health Care	Experimental	2	1	2	2	1	**8**

Hetlevik	2000	Int J Technol Assess Health Care	Experimental	2	1	2	2	0	**7**

Jousimaa	2002	Int J Technol Assess Health Care	Experimental	2	1	2	2	1	**8**

Kitahata	2003	Clinical Infectious Disease	Observational (before and after)	0	1	2	2	2	**7**

Kucher	2005	The New England Journal of medicine	Experimental	2	1	0	2	2	**7**

Lafata	2002	JGIM	Experimental	2	2	2	2	2	**10**

Lobach	1997	Am J Med	Experimental	2	0	2	2	2	**8**

Raebel	2005	Arch Intern Med	Experimental	2	1	2	2	2	**9**

McCowan	2001	Medical Informatics	Experimental	2	2	2	1	0	**7**

McMullin	2004	Annals of Family Medicine	Observational (retrospective cohort study)	0	1	0	2	2	**5**

Medow	2001	Medical Decision Making	Experimental	2	2	0	0	2	**6**

Meigs	2003	Diabetes Care	Experimental	2	1	2	2	2	**9**

Montgomery	2000	BMJ	Experimental	2	2	2	1	1	**8**

Mosen	2004	Chest	Observational (before and after)	0	2	2	2	2	**8**

Murtaugh	2005	Health Services Research	Experimental	2	1	2	1	2	**8**

Overhage	1996	Arch Intern Med	Experimental	1	2	2	2	2	**9**

Overhage	1997	JAMIA	Experimental	2	1	2	2	2	**9**

Poller	1993	J Clin Pathol	Experimental	2	2	1	2	2	**9**

Rood	2005	JAMIA	Experimental	2	2	1	2	2	**9**

Rossi	1997	JGIM	Experimental	2	1	2	2	2	**9**

Safran	1995	Lancet	Experimental	0	2	2	2	2	**8**

Schriger	1997	JAMA	Observational (interrupted time series)	1	1	2	2	2	**8**

Sequist	2005	JAMIA	Experimental	2	1	2	2	2	**9**

Shojonia	1998	JAMIA	Experimental	2	2	0	2	2	**8**

Steele	2005	American Journal of Preventive Medicine	Experimental	0	1	0	2	2	**5**

Thomas	1999	J Med Internet Res	Experimental	2	1	0	1	2	**6**

Tierney	2003	JGIM	Experimental	2	1	2	2	2	**9**

Turner	1994	Arch Intern Med	Experimental	2	1	2	2	1	**8**

Williams	1998	Arch Fam Med	Experimental	2	1	0	2	2	**7**

Zanetti	2003	Infection control and hospital epidemiology	Experimental	2	2	2	2	2	**10**

### Statistical Analysis

Each study, comparing the impact of CCG versus NCCG, was considered as a unit of analysis.

We estimated, within 95% confidence interval:

- **Positive Effect Prevalence**, calculated as the proportion of studies showing a positive effect of CCG on the total of selected studies.

- **Negative Effect Prevalence**, calculated as the proportion of studies not showing any or negative effect of CCG on the total of selected studies.

Chi-square test was performed in order to identify whether the differences between the proportions of the studies' positive and negative effects were statistically significant. The significance level was set at 5% (α = 0.05).

The effect of each specific feature on the process of care was also analysed in a backward logistic regression analysis which was carried out to evaluate the association of features with the positive effect of CCG, adjusting for the following variables:

• publication year, using 1999 (after publication of Shifman's article) as a cut-off year (1994-1999; 2000-2006);

• design of the study: observational and experimental studies;

• quality of the study, using 7 as cut-off score (5-7; 8-10)

Hosmer-Lemeshow test was applied to evaluate the goodness of fit of model. All analyses were carried out using SPSS package, version 13.0.

## Results

**Figure 1:**
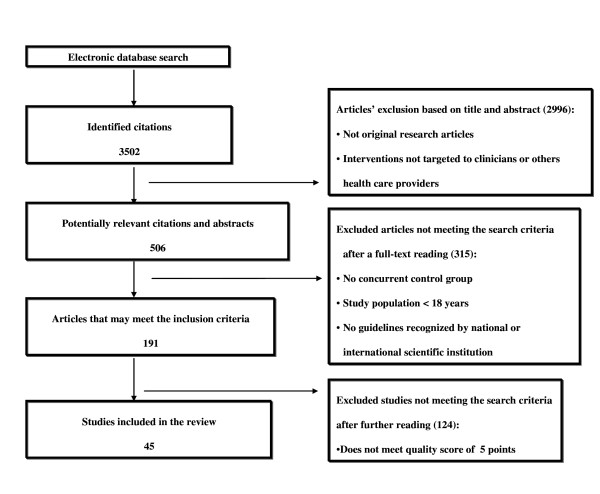
**Selection process of studies on computerized guidelines**.

A total number of 2,996 articles out of 3,502 was excluded because of the title and the content of abstract. Then, 191 out of 506 studies met the inclusion criteria. Forty-five articles were included in the final selection [14,15,22-64]. (Figure 1). Some of the articles included in Garg's and Kawamoto reviews [6,12] were excluded by our selection [see Additional file 1]. The characteristics of selected studies are shown in Table 3.

**Table 3:** Characteristics of selected studies.

Variables		Countries		
		**Europe**	**USA**	**Oceania**

**Study design**	Observational	2 (33.3%)	4 (66.7%)	0 (0.0%)
	Experimental	9 (23.1%)	29 (74.3%)	1 (2.6%)

**Type of patients**	Inpatient	2 (28.6%)	5 (71.4%)	0 (0.0%)
	Outpatient	4 (23.5%)	12 (70.6%)	1 (5.9%)

**Guidelines receivers**	Physicians	10(28.6%)	24 (68.6%)	1 (2.9%)
	Other care givers	1 (10.0%)	9 (90.0%)	0 (0%)

**Population of study**	Simulated	1 (25.0%)	3 (75.0%)	0 (0.0%)
	Real	10(24.4%)	30 (73.2%)	1 (3.3%)

**Type of centres involved in the study**	Non--academic	5 (22.7%)	17 (77.3%)	0 (0.0%)
	Academic	5 (25.0%)	15 (75.0%)	0 (0.0%)

**Number of centres involved in the study**	Multicentric	7 (36.8%)	11 (57.9%)	1 (5.3%)
	Monocentric	4 (15.4%)	22 (84.6%)	0 (0.0%)

**Type of guideline**	Preventive	1 (7.1%)	12 (85.7%)	1 (7.1%)
	Treatment	9 (32.1%)	19 (67.9%)	0 (0.0%)
	Both	1 (33.3%)	2 (66.7%)	0 (0.0%)

**Type of condition**	Acute	2 (28.6%)	5 (71.4)	0 (0.0%)
	Chronic	6 (22.2%)	21 (77.8%)	0 (0.0%)
	Both	3 (27.3%)	7 (63.6%)	1 (9.1%)

**Type of intervention**	Test or/and drugs	7 (29.2%)	17 (70.8%)	0 (0.0%)
	Other intervention	1 (14.3%)	6 (85.7%)	0 (0.0%)
	Both	3 (21.4%)	10 (71.4)	1 (7.1%)

*Automatic provision of recommendation in electronic version as part of clinician workflow *(proportion = 0.82) and *Degree of automation *(proportion = 0.80) were the most frequent features used in the CCG software described in the selected articles. On the contrary, the least frequent features were *Recommendation executed by noting agreement*, *Provision of a recommendation not just an assessment, Promotion of action rather than inaction *(proportion = 0.11) as shown in Table 1.

**Figure 2:**
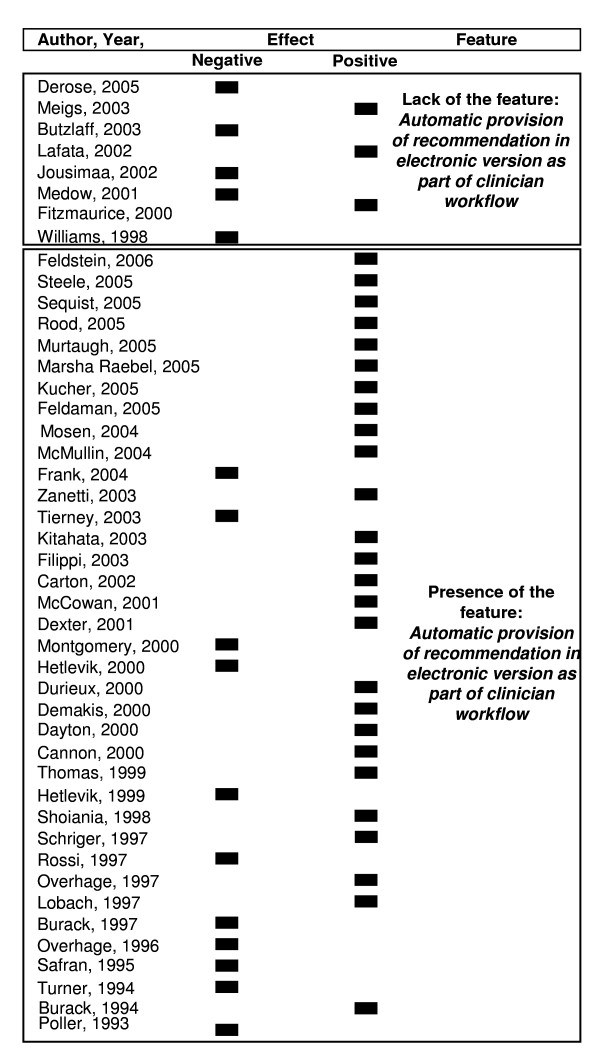
**Plot of the effect stratified by "*Automatic provision of recommendation in electronic version as part of clinician workflow" *feature**.

Proportions of studies with Positive and Negative Effect of CCG versus NCCG are shown in Figure 2. In the selected 45 articles the positive effect proportion of CCG was 0.64 (p = 0.053) [see Additional file 2].

The multivariable analysis highlighted two variables as statistically significant predictors of CCG positive impact on the process of care: *Automatic provision of recommendation in electronic version as part of clinician workflow *(Odds Ratio [OR]= 17.5; 95% confidence interval [CI]: 1.6-193.7) and *Publication Year *(OR = 6.7; 95%CI: 1.3-34.3). Besides, the feature *Justification of recommendation via provision of reasoning *(OR = 14.8; 95%CI: 0.9-224.2) resulted marginally significant in logistic analysis.

The goodness of fit of the logistic model was confirmed in the Hosmer-Lemeshow test (p = 0.905).

## Discussion

Previous researches [6,10,11] reviewed controlled clinical trials classified within different categories (e.g. drug dose determination, diagnosis, prevention) in order to assess the effects of CDSS on physician's performance and patient's outcomes. Enhancements on clinical performance were reported after the use of these tools. Furthermore, role of specific features of CDSS affecting clinical practice were identified by Kawamoto et al [12].

Our study instead focused on the effectiveness of CCG (a group of CDSS strictly related to the medical decision making). The functionality and the effectiveness of CCG until 1998 had been studied by Shiffman et al. [13]. They reviewed the literature showing that CCG delivered positive effect, but no quantitative and synthetic analysis were carried out. Our contribution provides an updated, systematic and quantitative analysis aiming to understand the design factors which are responsible for the success or the failure of computer-based guidelines compared with NCCG.

The resulting evidence showed that the use of CCG seems to have a significant impact on the process of care. In addition to qualitative evidence reported by Shiffman [13], the multivariable analysis highlighted the positive effect of the presence of an operating CCG system, characterized by the automatic provision of recommendation in electronic form as part of clinician workflow. This system is designed for providing automatic support to clinicians so that they don't need to look for computer advices. The system automatically provides support on clinical or administrative task and recommends execution or avoiding of it during the clinical process, (e.g. automatic recommendation of executing prophylaxis in patients at risk of deep-vein thrombosis [48]), and in decisions, such as the selection from a set of potential alternatives based on predefined criteria (e.g. automatic prompt of further assessment for potential Latent Tuberculosis Infection in patients selected according to specific criteria [38]) [65].

The positive effect might be related to time saving for clinicians, facilitation of the information retrieval and integration among different users.

The evidence of increased probability of positive effect for CCG, showed after 1999, might suggest that the improvement of the process of care may be related to the development of more automated CCG systems [66,67].

The physicians' involvement in decisions regarding clinical recommendations, even though marginally significant in the multivariable analysis, might be a key element for the effective organization of the whole process of care, relating to the improvement of the adherence of physicians to guidelines. This aspect is coherent to the active roles that physicians should play in Clinical Governance context [68,69].

Some limits of our study might be related to the lack of quantitative estimate of specific outcomes linked to clinical conditions. However, the evaluation of synthetic quantitative measures of CCG effect was unfeasible because of the high heterogeneity of analysed guidelines, population and outcomes. However, our work presents the synthetic result on the effectiveness of CCG, providing a quantitative and reproducible evaluation.

## Conclusions

Findings of this paper suggest clinicians, managers and other health care decision makers which features of CCG might improve the structure of an electronic system in health care settings. At the same time, the implementation of CCG may be integrated with more training and investment in user friendly hardware and software. Therefore, specific studies should be carried out to evaluate the cost-effectiveness of implementing CCG systems.

## Abbreviations

CCG: computerized clinical guidelines; CDSSs: computerized clinical decision support systems; NCCG: non-computerized clinical guidelines.

## Competing interests

The authors declare that they have no competing interests.

## Authors' contributions

GD, LP and WR contributed to the conception of this paper; GD, LP RG, LoS designed the study. SCC and RA selected articles that met the inclusion criteria and extracted data. LS, LP conducted the statistical analysis. All authors made substantial contributions to the interpretation of results and have seen and approved the final version.

## Pre-publication history

The pre-publication history for this paper can be accessed here:

http://www.biomedcentral.com/1472-6963/10/2/prepub

## Supplementary Material

Additional file 1 **Appendix 1**. Articles included in Garg and Kawamoto reviews and excluded in our review. Click here for file

Additional file 2 **Appendix 2**. List of outcomes of selected articles. Click here for file

## References

[B1] Field MJ Lohr KN eds Clinical practice guidelines: directions for a new program 1990 Washington, DC: National Academy Press 25144032

[B2] Shekelle P Eccles MP Grimshaw JM Woolf SH When should clinical guidelines be updated? BMJ 2001 323 7305 155 7 10.1136/bmj.323.7305.155 11463690 PMC1120790

[B3] Shekelle PG Woolf SH Eccles M Grimshaw J Clinical guidelines: developing guidelines BMJ 1999 318 7183 593 6 10037645 10.1136/bmj.318.7183.593 PMC1115034

[B4] Steinbrook R Guidance for guidelines N Engl J Med 2007 356 4 331 3 10.1056/NEJMp068282 17251529

[B5] Wyatt J Spiegelhalter D Clayton P Field trials of medical decision-aids: potential problems and solutions Proceedings of the Annual Symposium on Computer Application in Medical Care: 17-20 November 1991; Washington 1991 3 7 PMC2247484 1807610

[B6] Garg AX Adhikari NKJ McDonald H Rosas-Arellano MP Devereaux PJ Beyene J Sam J Haynes B Effects of Computerized Clinical Decision Support Systems on practitioner performance and patient outcomes JAMA 2005 293 1223 1238 10.1001/jama.293.10.1223 15755945

[B7] Shortliffe EH Computer programs to support clinical decision making JAMA 1987 258 1 61 6 10.1001/jama.258.1.61 3586293

[B8] Waitman LR Miller RA Pragmatics of implementing guidelines on the front lines J Am Med Inform Assoc 2004 11 5 436 8 10.1197/jamia.M1621 15449402 PMC516253

[B9] Wang D Peleg M Tu SW Shortliffe EH Greenes RA Representation of clinical practice guidelines for computer-based implementations Stud Health Technol Inform 2001 84 Pt 1 285 9 11604750

[B10] Hunt DL Haynes RB Hanna SE Smith K Effects of computer-based clinical decision support systems on physicians performance and patient outcomes J Am Med Assoc 1998 280 1339 1346 10.1001/jama.280.15.1339 9794315

[B11] Johnston ME Langton KB Haynes RB Mathieu A Effects of computer-based clinical decision support systems on clinician performance and patient outcome. A critical appraisal of research Ann Intern Med 1994 120 2 135 42 8256973 10.7326/0003-4819-120-2-199401150-00007

[B12] Kawamoto K Houlihan CA Balas EA Lobach DF Improving clinical practice using clinical decision support systems: a systematic review of trials to identify features critical to success BMJ 2005 330 765 773 10.1136/bmj.38398.500764.8F 15767266 PMC555881

[B13] Shiffman RN Liaw Y Brandt CA Corb GJ Computer-based guideline implementation system: a systematic review of functionality and effectiveness JAMIA 1999 6 104 114 10094063 10.1136/jamia.1999.0060104 PMC61349

[B14] Overhage JM Tierney WM McDonald CJ Computer reminders to implement preventive care guidelines for hospitalized patients Arch Intern Med 1996 156 1551 1556 10.1001/archinte.156.14.1551 8687263

[B15] Raebel MA Lyons EE Chester EA Bodily MA Kelleher JA Long CL Miller C Magid DJ Improving laboratory monitoring at initiation of drug therapy in ambulatory care Arch Intern Med 2005 165 2395 2401 10.1001/archinte.165.20.2395 16287769

[B16] Cabana MD Rand CS Becher OJ Rubin HR Reasons for pediatrician nonadherence to asthma guidelines Arch Pediatr Adolesc Med 2001 155 9 1057 62 11529809 10.1001/archpedi.155.9.1057

[B17] Cabana MD Ebel BE Cooper-Patrick L Powe NR Rubin HR Rand CS Barriers pediatricians face when using asthma practice guidelines Arch Pediatr Adolesc Med 2000 54 7 685 93 10.1001/archpedi.154.7.685 10891020

[B18] Weinberger M Seventeen Years of Asthma Guidelines: why Hasn't the Outcome Improved for Children? J Pediatr 2009 54 6 786 8 10.1016/j.jpeds.2009.01.003 19446095

[B19] Haynes RB Walker C Computer-aided quality assurance Arch Intern Med 1987 147 1297 1301 10.1001/archinte.147.7.1297 3606287

[B20] Damiani G Pinnarelli L Sammarco A Sommella L Francucci M Ricciardi W Postoperative Pulmonary Function inOpen versus Laparoscopic Cholecystectomy: A Meta-Analysis of the Tiffenau Index Dig Surg 2008 25 1 7 10.1159/000114193 18235188

[B21] Chalmers TC Smith H Jr Blackburn B Silverman B Schroeder B Reitman D Ambroz A method for assessing the quality of a randomized control trial A Control Clin Trials 1981 2 1 31 49 10.1016/0197-2456(81)90056-8 7261638

[B22] Jousimaa J Makela M Kunnamo I MacLennan G Grimsha JM Primary care guidelines on consultation practices: the effectiveness of computerized versus paper-based versions Int J Technol Assess Health Care 2002 18 586 596 12391951

[B23] Rood E Bossman RJ Spoel JI Van Der Taylor P Zandstra DF Use of a computerized guideline for glucose regulation in the intensive care unit improved both guideline adherence and glucose regulation JAMIA 2005 12 172 180 15561795 10.1197/jamia.M1598 PMC551549

[B24] Butzlaff M Vollmar HC Floer B Koneczny N Isfort J Lange S Learning with computerized guidelines in general practice? A randomized controlled trial Fam Pract 2003 21 183 188 10.1093/fampra/cmh214 15020389

[B25] Medow MA Wilt TJ Dysken S Hillson SD Woods S Borowsky SJ Effect of written and computerized decision support aids for the U.S. Agency for Health Care Policy and Research depression guidelines on the evaluation of hypothetical clinical scenarious Med Decis Making 2001 21 344 356 11575484 10.1177/0272989X0102100501

[B26] Durieux P Nizard R Ravaud P Mounier N Lepage E A Clinical Decision Support System for prevention of venous thromboembolism. Effect on physician behaviour JAMA 2000 283 2816 2821 10.1001/jama.283.21.2816 10838650

[B27] Rossi RA Every NR A computerized intervention to decrease the use of calcium channel blockers in hypertension J Gen Intern Med 1997 12 672 678 10.1046/j.1525-1497.1997.07140.x 9383135 PMC1497186

[B28] Safran C Rind DM Davis RB Ives D Sands DZ Currier J Slack WV Makadon HJ Cotton DJ Guidelines for management of HIV infection with compute-based patient's record Lancet 1995 346 341 346 10.1016/S0140-6736(95)92226-1 7623532

[B29] Dexter PR Perkins S Overhage M Maharry K Kohler RB McDonald CJ A computerized reminder system to increase the use of preventive care for hospitalized patients N Engl J Med 2001 345 965 970 10.1056/NEJMsa010181 11575289

[B30] Mosen D Elliott CG Egger MJ Mundorff M Hopkins J Patterson R Gardner RM The effect of a computerized reminder system on the prevention of postoperative venous thromboembolism Chest 2004 125 1635 1641 10.1378/chest.125.5.1635 15136370

[B31] Shojania KG Yokoe D Platt R Fiskio J Ma'luf N Bates DW Reducing vancomycin use utilizing a computer guideline: results of a randomized controlled trial JAMIA 1998 5 554 562 9824802 10.1136/jamia.1998.0050554 PMC61335

[B32] Schriger DL Baraff LJ Rogers WH Cretin S Implementing of clinical guidelines using a computer charting system. Effect on the initial care of health care workers exposed to body fluids JAMA 1997 278 1585 1590 10.1001/jama.278.19.1585 9370504

[B33] Thomas KW Dayton CS Peterson MW Evaluation of internet-based clinical decision support systems JMIR 1999 1 6 36 10.2196/jmir.1.2.e6 PMC1761710 11720915

[B34] Carton M Auvert B Guerini H Boulard JC Heautot JF Landre MF Beauchet A Sznajderi M Brun-Ney D Chagnon S Assessment of radiological referral practice and effect of computer-based guidelines on radiological requests in two emergency departments Clin Radiol 2002 57 123 128 10.1053/crad.2001.0827 11977945

[B35] Lobach DF Hammond WE Computerized decision support based on a clinical practice guideline improves compliance with care standards Am J Med 1997 102 89 98 10.1016/S0002-9343(96)00382-8 9209205

[B36] Tierney WM Overhage JM Murray MD Harris LE Zhou XH Eckert GJ Smith FE Nienaber N McDonald CJ Wolinsky FD Effects of computerized guidelines for managing heart disease in primary care J Gen Intern Med 2003 18 967 976 10.1111/j.1525-1497.2003.30635.x 14687254 PMC1494965

[B37] McMullin ST Lonergan TP Rynearson CS Doerr TD Veregge PA Scanlan ES Impact of an evidence-based computerized decision support system on primary care prescription costs Ann Fam Med 2004 2 494 498 10.1370/afm.233 15506587 PMC1466719

[B38] Steele AW Eisert S Davidson A Sandison T Lyons P Garrett N Gabow P Ortiz E Using computerized clinical decision support for latent tuberculosis infection screening Am J Prev Med 2005 28 281 284 10.1016/j.amepre.2004.12.012 15766616

[B39] Dayton CS Ferguson S Hornick DB Peterson MW Evaluation of an internet-based decision-support system for applying the ATS/CDC guidelines for tuberculosis preventive therapy Med Decis Making 2000 20 1 6 10.1177/0272989X0002000101 10638531

[B40] Overhage JM Tierney WM Zhou XA McDonald CJ A randomized trial of "corollary orders" to prevent errors of omission JAMIA 1997 4 364 375 9292842 10.1136/jamia.1997.0040364 PMC61254

[B41] Filippi A Sabatini A Badioli L Samani F Mazzaglia G Catapano A Cricelli C Effects of an automated electronic reminder in changing the antiplatelet drug-prescribing behavior among italian general practitioners in diabetic patients Diabetes Care 2003 26 1497 1500 10.2337/diacare.26.5.1497 12716811

[B42] Turner RC Peden JG O'Brien K Patient-carried card prompts vs computer-generated prompts to remind private practice physicians to perform health maintenance measures Arch Intern Med 1994 154 1957 1960 10.1001/archinte.154.17.1957 8074599

[B43] Burack RC Phyllis AG Promoting screening mammography in inner-city settings. The sustained effectiveness of computerized reminders in a randomized controlled trial Med Care 1997 35 921 931 10.1097/00005650-199709000-00005 9298081

[B44] Burack RC Phyllis AG George J Stengle W Warbasse L Moncrease A Promoting screening mammography in inner-city settings: a randomized controlled trial of computerized reminders as a component of a program to facilitate mammography Med Care 1994 32 609 624 10.1097/00005650-199406000-00006 8189778

[B45] Sequist TD Gandhi TK Karson AS Fiskio JM Bugbee D Sperling M Cook EF Orav EJ Fairchild DJ Bates DW A randomized trial of electronic clinical reminders to improve quality of care for diabetes and coronary artery disease JAMIA 2005 12 431 437 15802479 10.1197/jamia.M1788 PMC1174888

[B46] Derose SF Dudl JR Benson VM Contreas R Nakahiro RK Ziel FH Point-of-service reminders for prescribing cardiovascular medications Am J Manag Care 2005 11 298 304 15898218

[B47] Kitahata MM Dillingham PW Chaiyakunapruk N Buskin SE Jones JL Harrington RD Hooton TM Holmes KK Electronic human immunodeficiency virus (HIV) clinical reminder system improves adherence to practice guidelines among the University of Washington HIV study cohort Clin Infect Dis 2003 36 803 811 10.1086/368085 12627367

[B48] Kucher N Koo S Quiroz R Cooper JM Paterno MD Soukonnikov B Goldhaber SZ Electronic alerts to prevent venous thromboembolism among hospitalized patients N Engl J Med 2005 352 969 977 10.1056/NEJMoa041533 15758007

[B49] Feldman PH Murtaugh CM Pezzin LE McDonald MV Peng TR Just-in-time evidence-based e-mail "reminders" in home health care: impact on patient outcomes Health Serv Res 2005 40 865 885 10.1111/j.1475-6773.2005.00389.x 15960695 PMC1361172

[B50] Frank O Litt J Beilby J Opportunistic electronic reminders Aust Fam Physician 2004 33 87 90 14988972

[B51] Feldstein A Elmer PJ Smith DH Herson M Orwoll E Chen C Aickin M Swain MC Electronic medical record reminder improves osteoporosis management after a fracture: a randomized, controlled trial J Am Geriatr Soc 2006 54 450 457 10.1111/j.1532-5415.2005.00618.x 16551312

[B52] Williams RB Boles M Johnson RE A patient-initiated system for preventive health care Arch Fam Med 1998 7 338 345 10.1001/archfami.7.4.338 9682687

[B53] Zanetti G Flanagan HL Cohn LH Giardina R Platt R Improvement of intraoperative antibiotic prophylaxis in prolonged cardiac surgery by automated alerts in the operating room Infect Control Hosp Epidemiol 2003 24 13 16 10.1086/502109 12558230

[B54] Demakis JG Beauchamp C Cull WL Denwood R Eisen SA Lofgren R Nichol K Woolliscroft J Henderson WG Improving residents' compliance with standards of ambulatory care JAMA 2000 284 1411 1416 10.1001/jama.284.11.1411 10989404

[B55] Hetlevik I Holmen J Kruger O Implementing clinical guidelines in the treatment of hypertension in general practice Scand J Prim Health Care 1999 17 35 40 10.1080/028134399750002872 10229991

[B56] Hetlevik I Holmen J Kruger O Kristensen P Iversen H Furuseth K Implementing clinical guidelines in the treatment of diabetes mellitus in general practice Int J Technol Assess Health Care 2000 16 210 227 10.1017/S0266462300161185 10815366

[B57] Murtaugh CM Pezzin LE McDonald MV Feldman PH Peng TR Just-in-time evidence-based e-mail "reminders" in home health care: impact on nurse practices Health Serv Res 2005 40 849 864 10.1111/j.1475-6773.2005.00388.x 15960694 PMC1361171

[B58] Lafata JE Baker AM Divine GW McCarthy BD Xi H The use of computerized birthday greeting reminders in the management of diabetes J Gen Intern Med 2002 17 521 530 10.1046/j.1525-1497.2002.10901.x 12133142 PMC1495071

[B59] Fitzmaurice DA Hobbs FDR Murray ET Holder RL Allan TF Rose PE Oral anticoagulation management in primary care with the use of computerized decision support and near-patient testing Arch Intern Med 2000 160 2343 2348 10.1001/archinte.160.15.2343 10927732

[B60] Poller L Wright D Rowlands M rospective comparative study of computer programs used for management of warfarin J Clin Pathol 1993 46 299 303 10.1136/jcp.46.4.299 8496384 PMC501207

[B61] Meigs JB Cagliero E Dubey A Murphy-Sheehy P Gildesgame C Chueh H Barry M Singer DE Nathan MD A controlled trial of web-based diabetes disease management Diabetes Care 2003 26 750 757 10.2337/diacare.26.3.750 12610033

[B62] Montgomery AA Fahey T Peters TJ MacIntosh C Sharp DJ Evaluation of computer based clinical decision support system and risk chart for management of hypertension in primary care: randomised controlled trial BMJ 2000 320 686 690 10.1136/bmj.320.7236.686 10710578 PMC27312

[B63] Cannon DS Allen SN A comparison of the effects of computer and manual reminders on compliance with a mental health clinical practice guideline JAMIA 2000 7 196 203 10730603 10.1136/jamia.2000.0070196 PMC61473

[B64] McCowan C Neville RG Ricketts W Warner FC Hoskins G Thomas GE Lessons from a randomized controlled trial designed to evaluate computer decision support software to improve the management of asthma Med Inform 2001 26 3 191 201 10.1080/14639230110067890 11706929

[B65] Wang D Peleg M Tu SW Boxwala AA Greenes RA Patel VL Shortliffe EH Representation primitives, process models and patient data in computer-interpretable clinical practice guidelines: a literature review of guideline representation models Int J Med Inform 2002 68 1-3 59 70 10.1016/S1386-5056(02)00065-5 12467791

[B66] Isern D Moreno A Computer-based execution of clinical guidelines: a review Int J Med Inform 2008 77 12 787 808 10.1016/j.ijmedinf.2008.05.010 18639485

[B67] Goldstein MK Using health information technology to improve hypertension management Curr Hypertens Rep 2008 10 3 201 7 10.1007/s11906-008-0038-6 18765090

[B68] Scally G Donaldson LJ Clinical governance and the drive for quality improvement in the new NHS in England BMJ 1998 317 61 65 9651278 10.1136/bmj.317.7150.61 PMC1113460

[B69] Halligan A Donaldson LJ Implementing clinical governance: turning vision into reality BMJ 2001 322 1413 1417 10.1136/bmj.322.7299.1413 11397753 PMC1120478

