# Supplementary material for: The effectiveness of computerized clinical guidelines in the process of care: a systematic review
Source: BMC Health Serv Res. 2010 Jan 4;10:2. doi: 10.1186/1472-6963-10-2 (PMC2837004; doi:10.1186/1472-6963-10-2)
Supplement: Additional file 1 — Appendix 1. Articles included in Garg and Kawamoto reviews and excluded in our review. [file 1472-6963-10-2-S1.DOC]

APPENDIX 1

Articles included in **Garg** review and excluded in our review

Reasons for exclusion: lacking of a certified computerized guidelines implemented in the assessed CDSS compared with non-computerized guidelines.

1. Schriger DL, Gibbons PS, Langone CA, Lee S, Altshuler LL. Enabling the diagnosis of occult psychiatric illness in the emergency department: a randomized, controlled trial of the computerized, self-administered PRIME-MD diagnostic system. Ann Emerg Med. 2001;37:132-140. FULL TEXT | ISI | PUBMED
2. Rollman BL, Hanusa BH, Lowe HJ, et al. A randomized trial using computerized decision support to improve treatment of major depression in primary care. J Gen Intern Med. 2002;17:493-503. FULL TEXT | ISI | PUBMED
3. Selker HP, Beshansky JR, Griffith JL, et al. Use of the acute cardiac ischemia time-insensitive predictive instrument (ACI-TIPI) to assist with triage of patients with chest pain or other symptoms suggestive of acute cardiac ischemia: a multicenter, controlled clinical trial. Ann Intern Med. 1998;129:845-855. FREE FULL TEXT
4. Wellwood J, Johannessen S, Spiegelhalter DJ. How does computer-aided diagnosis improve the management of acute abdominal pain? Ann R Coll Surg Engl. 1992;74:40-46. ISI | PUBMED
5. Bogusevicius A, Maleckas A, Pundzius J, Skaudickas D. Prospective randomised trial of computer-aided diagnosis and contrast radiography in acute small bowel obstruction. Eur J Surg. 2002;168:78-83. FULL TEXT | ISI | PUBMED
6. Burack RC, Gimotty PA, George J, Simon MS, Dews P, Moncrease A. The effect of patient and physician reminders on use of screening mammography in a health maintenance organization: results of a randomized controlled trial. Cancer. 1996;78:1708-1721. FULL TEXT | ISI | PUBMED
7. Burack RC, Gimotty PA, Simon M, et al. The effect of adding Pap smear information to a mammography reminder system in an HMO: results of randomized controlled trial. Prev Med. 2003;36:547-554. FULL TEXT | ISI | PUBMED
8. Tang PC, LaRosa MP, Newcomb C, Gorden SM. Measuring the effects of reminders for outpatient influenza immunizations at the point of clinical opportunity. J Am Med Inform Assoc. 1999;6:115-121. FREE FULL TEXT
9. Tape TG, Campbell JR. Computerized medical records and preventive health care: success depends on many factors. Am J Med. 1993;94:619-625. FULL TEXT | ISI | PUBMED
10. Frame PS, Zimmer JG, Werth PL, et al. Computer-based vs manual health maintenance tracking: a controlled trial. Arch Fam Med. 1994;3:581-588. FREE FULL TEXT
11. Bonevski B, Sanson-Fisher RW, Campbell E, Carruthers A, Reid AL, Ireland M. Randomized controlled trial of a computer strategy to increase general practitioner preventive care. Prev Med. 1999;29:478-486. FULL TEXT | ISI | PUBMED
12. Montori VM, Dinneen SF, Gorman CA, et al. The impact of planned care and a diabetes electronic management system on community-based diabetes care. Diabetes Care. 2002;25:1952-1957. FREE FULL TEXT
13. Lowensteyn I, Joseph L, Levinton C, Abrahamowicz M, Steinert Y, Grover S. Can computerized risk profiles help patients improve their coronary risk? the results of the Coronary Health Assessment Study (CHAS). Prev Med. 1998;27:730-737. FULL TEXT | ISI | PUBMED
14. Selker HP, Beshansky JR, Griffith JL. Use of the electrocardiograph-based thrombolytic predictive instrument to assist thrombolytic and reperfusion therapy for acute myocardial infarction: a multicenter, randomized, controlled, clinical effectiveness trial. Ann Intern Med. 2002;137:87-95. FREE FULL TEXT
15. Ansari M, Shlipak MG, Heidenreich PA, et al. Improving guideline adherence: a randomized trial evaluating strategies to increase beta-blocker use in heart failure. Circulation. 2003;107:2799-2804. FREE FULL TEXT
16. Weir CJ, Lees KR, MacWalter RS, et al. Cluster-randomized, controlled trial of computer-based decision support for selecting long-term anti-thrombotic therapy after acute ischaemic stroke. QJM. 2003;96:143-153. FREE FULL TEXT
17. Murray MD, Harris LE, Overhage JM, et al. Failure of computerized treatment suggestions to improve health outcomes of outpatients with uncomplicated hypertension: results of a randomized controlled trial. Pharmacotherapy. 2004;24:324-337. FULL TEXT | ISI | PUBMED
18. Rubenstein LV, McCoy JM, Cope DW, et al. Improving patient quality of life with feedback to physicians about functional status. J Gen Intern Med. 1995;10:607-614. ISI | PUBMED
19. Dexter PR, Wolinsky FD, Gramelspacher GP, et al. Effectiveness of computer-generated reminders for increasing discussions about advance directives and completion of advance directive forms: a randomized, controlled trial. Ann Intern Med. 1998;128:102-110. FREE FULL TEXT
20. Kuperman GJ, Teich JM, Tanasijevic MJ, et al. Improving response to critical laboratory results with automation: results of a randomized controlled trial. J Am Med Inform Assoc. 1999;6:512-522. FREE FULL TEXT
21. Eccles M, McColl E, Steen N, et al. Effect of computerised evidence based guidelines on management of asthma and angina in adults in primary care: cluster randomised controlled trial. BMJ. 2002;325:941. FREE FULL TEXT
22. Lesourd F, Avril C, Boujennah A, Parinaud J. A computerized decision support system for ovarian stimulation by gonadotropins. Fertil Steril. 2002;77:456-460. FULL TEXT | ISI | PUBMED
23. Fihn SD, McDonell MB, Vermes D, et al. A computerized intervention to improve timing of outpatient follow-up: a multicenter randomized trial in patients treated with warfarin. J Gen Intern Med. 1994;9:131-139. ISI | PUBMED
24. Tierney WM, Miller ME, Overhage JM, McDonald CJ. Physician inpatient order writing on microcomputer workstations: effects on resource utilization. JAMA. 1993;269:379-383. FREE FULL TEXT
25. Shea S, Sideli RV, DuMouchel W, Pulver G, Arons RR, Clayton PD. Computer-generated informational messages directed to physicians: effect on length of hospital stay. J Am Med Inform Assoc. 1995;2:58-64. FREE FULL TEXT
26. Bates DW, Kuperman GJ, Rittenberg E, et al. A randomized trial of a computer-based intervention to reduce utilization of redundant laboratory tests. Am J Med. 1999;106:144-150. FULL TEXT | ISI | PUBMED
27. Fitzmaurice DA, Hobbs FD, Murray ET, Bradley CP, Holder R. Evaluation of computerized decision support for oral anticoagulation management based in primary care. Br J Gen Pract. 1996;46:533-535. ISI | PUBMED
28. Vadher B, Patterson DL, Leaning M. Evaluation of a decision support system for initiation and control of oral anticoagulation in a randomised trial. BMJ. 1997;314:1252-1256. FREE FULL TEXT
29. Vadher BD, Patterson DL, Leaning M. Comparison of oral anticoagulant control by a nurse-practitioner using a computer decision-support system with that by clinicians. Clin Lab Haematol. 1997;19:203-207. FULL TEXT | ISI | PUBMED
30. Ageno W, Turpie AG. A randomized comparison of a computer-based dosing program with a manual system to monitor oral anticoagulant therapy. Thromb Res. 1998;91:237-240. FULL TEXT | ISI | PUBMED
31. Poller L, Shiach CR, MacCallum PK, et al. Multicentre randomised study of computerised anticoagulant dosage. Lancet. 1998;352:1505-1509. FULL TEXT | ISI | PUBMED
32. Manotti C, Moia M, Palareti G, Pengo V, Ria L, Dettori AG. Effect of computer-aided management on the quality of treatment in anticoagulated patients: a prospective, randomized, multicenter trial of APROAT (Automated PRogram for Oral Anticoagulant Treatment). Haematologica. 2001;86:1060-1070. FREE FULL TEXT
33. Mungall DR, Anbe D, Forrester PL, et al. A prospective randomized comparison of the accuracy of computer-assisted versus GUSTO nomogram–directed heparin therapy. Clin Pharmacol Ther. 1994;55:591-596. ISI | PUBMED
34. Verner D, Seligmann H, Platt S, et al. Computer assisted design of a theophylline dosing regimen in acute bronchospasm: serum concentrations and clinical outcome. Eur J Clin Pharmacol. 1992;43:29-33. FULL TEXT | ISI | PUBMED
35. Casner PR, Reilly R, Ho H. A randomized controlled trial of computerized pharmacokinetic theophylline dosing versus empiric physician dosing. Clin Pharmacol Ther. 1993;53:684-690. ISI | PUBMED
36. Ryff-de Leche A, Engler H, Nutzi E, Berger M, Berger W. Clinical application of two computerized diabetes management systems: comparison with the log-book method. Diabetes Res. 1992;19:97-105. ISI | PUBMED
37. Horn W, Popow C, Miksch S, et al. Development and evaluation of VIE-PNN, a knowledge-based system for calculating the parenteral nutrition of newborn infants. Artif Intell Med. 2002;24:217-228. FULL TEXT | ISI | PUBMED
38. Rotman BL, Sullivan AN, McDonald TW, et al. A randomized controlled trial of a computer-based physician workstation in an outpatient setting: implementation barriers to outcome evaluation. J Am Med Inform Assoc. 1996;3:340-348. FREE FULL TEXT
39. Tamblyn R, Huang A, Perreault R, et al. The medical office of the 21st century (MOXXI): effectiveness of computerized decision-making support in reducing inappropriate prescribing in primary care. CMAJ. 2003;169:549-556. FREE FULL TEXT

Reason for exclusion: articles contained in proceedings

1. Flanagan JR, Doebbeling BN, Dawson J, Beekmann S. Randomized study of online vaccine reminders in adult primary care. Proc AMIA Symp. 1999:755-759. PUBMED
2. Nilasena DS, Lincoln MJ. A computer-generated reminder system improves physician compliance with diabetes preventive care guidelines. Proc Annu Symp Comput Appl Med Care. 1995:640-645. PUBMED
3. Petrucci K, Petrucci P, Canfield K, et al. Evaluation of UNIS: urological nursing information systems. Proc Annu Symp Comput Appl Med Care. 1991:43-47. PUBMED
4. East TD, Heermann LK, Bradshaw RL, et al. Efficacy of computerized decision support for mechanical ventilation: results of a prospective multi-center randomized trial. Proc AMIA Symp. 1999:251-255. PUBMED
5. Hales JW, Gardner RM, Jacobson JT. Factors impacting the success of computerized preadmission screening. Proc Annu Symp Comput Appl Med Care. 1995:728-732. PUBMED

Reason for exclusion: articles focused on pediatric patients

1. Christakis DA, Zimmerman FJ, Wright JA, et al. A randomized controlled trial of point-of-care evidence to improve the antibiotic prescribing practices for otitis media in children. Pediatrics. 2001;107:E15.

Articles included in **Kawamoto** Review and excluded in our review

Reasons for exclusion: lacking of a certified computerized guidelines implemented in the assessed CDSS compared with non- computerized guidelines.

1. Frame PS, Zimmer JG, Werth PL, Hall WJ, Eberly SW. Computer-based vs manual health maintenance tracking. A controlled trial. *Arch Fam Med*. 1994;3:581-588.
2. Dexter PR, Wolinsky FD, Gramelspacher GP, et al. Effectiveness of computer-generated reminders for increasing discussions about advance directives and completion of advance directive forms. A randomized, controlled trial. *Ann Intern Med*. 1998;128:102-110.
3. Eccles M, McColl E, Steen N, et al. Effect of computerised evidence based guidelines on management of asthma and angina in adults in primary care: cluster randomised controlled trial. *BMJ*. 2002;325:941-947.
4. Litzelman DK, Dittus RS, Miller ME, Tierney WM. Requiring physicians to respond to computerized reminders improves their compliance with preventive care protocols. *J Gen Intern Med*. 1993;8:311-317.
5. Tierney WM, Miller ME, Overhage JM, McDonald CJ. Physician inpatient order writing on microcomputer workstations. Effects on resource utilization. *JAMA*. 1993;269:379-383.
6. Harpole LH, Khorasani R, Fiskio J, Kuperman GJ, Bates DW. Automated evidence-based critiquing of orders for abdominal radiographs: impact on utilization and appropriateness. *J Am Med Inform Assoc*. 1997;4:511-521.
7. Kuperman GJ, Teich JM, Tanasijevic MJ, et al. Improving response to critical laboratory results with automation: results of a randomized controlled trial. *J Am Med Inform Assoc*. 1999;6:512-522.
8. White P, Atherton A, Hewett G, Howells K. Using information from asthma patients: a trial of information feedback in primary care. *BMJ*. 1995;311:1065-1069.
9. Buchsbaum DG, Buchanan RG, Lawton MJ, Elswick RK Jr, Schnoll SH. A program of screening and prompting improves short-term physician counseling of dependent and nondependent harmful drinkers. *Arch Intern Med*. 1993;153:1573-1577.
10. Stamos TD, Shaltoni H, Girard SA, Parrillo JE, Calvin JE. Effectiveness of chart prompts to improve physician compliance with the National Cholesterol Education Program guidelines. *Am J Cardiol*. 2001;88:1420-1423.
11. Leviton LC, Goldenberg RL, Baker CS, et al. Methods to encourage the use of antenatal corticosteroid therapy for fetal maturation: a randomized controlled trial. *JAMA*. 1999;281:46-52.
12. Gimotty PA, Burack RC, George JA. Delivery of preventive health services for breast cancer control: a longitudinal view of a randomized controlled trial. *Health Serv Res*. 2002;37:65-85.
13. Burack RC, Gimotty PA, George J, Simon MS, Dews P, Moncrease A. The effect of patient and physician reminders on use of screening mammography in a health maintenance organization. Results of a randomized controlled trial. *Cancer*. 1996;78:1708-1721.
14. Rosser WW, Hutchison BG, McDowell I, Newell C. Use of reminders to increase compliance with tetanus booster vaccination. *CMAJ*. 1992;146:911-917.
15. Rosser W, McDowell I. Documenting smoking status. *Can Fam Physician*. 1992;38:1623-1628.
16. McDonald CJ, Hui SL, Tierney WM. Effects of computer reminders for influenza vaccination on morbidity during influenza epidemics. *MD Comput*. 1992;9:304-312.
17. Williams RB, Boles M, Johnson RE. A patient-initiated system for preventive health care. A randomized trial in community-based primary care practices. *Arch Fam Med*. 1998;7:338-345.
18. Burack RC, Gimotty PA, George J, et al. How reminders given to patients and physicians affected pap smear use in a health maintenance organization: results of a randomized controlled trial. *Cancer*. 1998;82:2391-2400.
19. Bankhead C, Richards SH, Peters TJ, et al. Improving attendance for breast screening among recent non-attenders: a randomised controlled trial of two interventions in primary care. *J Med Screen*. 2001;8:99-105.
20. Dowrick C. Does testing for depression influence diagnosis or management by general practitioners? *Fam Pract*. 1995;12:461-465.
21. Dowrick C, Buchan I. Twelve month outcome of depression in general practice: Does detection or disclosure make a difference? *BMJ*. 1995;311:1274-1276.
22. Hay WI, van Ineveld C, Browne G, et al. Prospective care of elderly patients in family practice. Is screening effective? *Can Fam Physician*. 1998;44:2677-2687.
23. Somkin CP, Hiatt RA, Hurley LB, Gruskin E, Ackerson L, Larson P. The effect of patient and provider reminders on mammography and Papanicolaou smear screening in a large health maintenance organization. *Arch Intern Med*. 1997;157:1658-1664.
24. Ray WA, Stein CM, Byrd V, et al. Educational program for physicians to reduce use of non-steroidal anti-inflammatory drugs among community-dwelling elderly persons: a randomized controlled trial. *Med Care*. 2001;39:425-435.
25. Pritchard DA, Straton JA, Hyndman J. Cervical screening in general practice. *Aust J Public Health*. 1995;19:167-172.
26. van Wijk MA, van der Lei J, Mosseveld M, Bohnen AM, van Bemmel JH. Assessment of decision support for blood test ordering in primary care. A randomized trial. *Ann Intern Med*. 2001;134:274-281.
27. Bates DW, Kuperman GJ, Rittenberg E, et al. A randomized trial of a computer-based intervention to reduce utilization of redundant laboratory tests. *Am J Med*. 1999;106:144-150.
28. Rotman BL, Sullivan AN, McDonald TW, et al. A randomized controlled trial of a computer-based physician workstation in an outpatient setting: implementation barriers to outcome evaluation. *J Am Med Inform Assoc*. 1996;3:340-348.
29. Rascati KL, Okano GJ, Burch C. Evaluation of physician intervention letters. *Med Care*. 1996;34:760-766.
30. Okano GJ, Rascati KL. Effects of Medicaid drug utilization review intervention letters. *Clin Ther*. 1995;17:525-533.
31. Connors AF, Dawson NV, Desbiens NA, et al, for the SUPPORT Principal Investigators. A controlled trial to improve care for seriously ill hospitalized patients. The study to understand prognoses and preferences for outcomes and risks of treatments (SUPPORT). *JAMA*. 1995;274:1591-1598.
32. Desbiens NA, Wu AW, Yasui Y, et al. Patient empowerment and feedback did not decrease pain in seriously ill hospitalized adults. *Pain*. 1998;75:237-246.
33. Desbiens NA, Wu AW. Pain and suffering in seriously ill hospitalized patients. *J Am Geriatr Soc*. 2000;48:S183-S186.
34. Simon GE, VonKorff M, Rutter C, Wagner E. Randomised trial of monitoring, feedback, and management of care by telephone to improve treatment of depression in primary care. *BMJ*. 2000;320:550-554.
35. Frolich A. Combined reports on serum calcium and discriminant functions increase the diagnostic rate of hypercalcaemia. *Scand J Clin Lab Invest*. 1997;57:725-729.
36. Mazonson PD, Mathias SD, Fifer SK, Buesching DP, Malek P, Patrick DL. The mental health patient profile: does it change primary care physicians’ practice patterns? *J Am Board Fam Pract*. 1996;9:336-345.
37. Mathias SD, Fifer SK, Mazonson PD, Lubeck DP, Buesching DP, Patrick DL. Necessary but not sufficient: the effect of screening and feedback on outcomes of primary care patients with untreated anxiety. *J Gen Intern Med*. 1994;9:606-615.
38. Gans KM, Lapane KL, Lasater TM, Carleton RA. Effects of intervention on compliance to referral and lifestyle recommendations given at cholesterol screening programs. *Am J Prev Med*. 1994;10:275-282.
39. Selker HP, Beshansky JR, Griffith JL, for the TPI Trial Investigators. Use of the electrocardiograph-based thrombolytic predictive instrument to assist thrombolytic and reperfusion therapy for acute myocardial infarction. A multicenter, randomized, controlled, clinical effectiveness trial. *Ann Intern Med*. 2002;137:87-95.

Reason for exclusion: articles contained in proceedings

1. Lobach DF. Electronically distributed, computer-generated, individualized feedback enhances the use of a computerized practice guideline. *Proc AMIA Symp*. 1996;493-497.
2. Lobach DF, Hammond WE. Development and evaluation of a Computer-Assisted Management Protocol (CAMP): improved compliance with care guidelines for diabetes mellitus. *Proc Annu Symp Comput Appl Med Care*. 1994;787-791.
3. Nilasena DS, Lincoln MJ. A computer-generated reminder system improves physician compliance with diabetes preventive care guidelines. *Proc Annu Symp Comput Appl Med Care*. 1995;640-645.
4. Flanagan JR, Doebbeling BN, Dawson J, Beekmann S. Randomized study of online vaccine reminders in adult primary care. *Proc AMIA Symp*. 1999;755-759.
5. Bates DW, Kuperman GJ, Rittenberg E, et al. Reminders for redundant tests: results of a randomized controlled trial. *Proc Annu Symp Comput Appl Med Care*. 1995;935.

Reason for exclusion: articles focused on pediatric patients

1. Christakis DA, Zimmerman FJ, Wright JA, Garrison MM, Rivara FP, Davis RL. A randomized controlled trial of point-of-care evidence to improve the antibiotic prescribing practices for otitis media in children. *Pediatrics*. 2001;107:E15-E18.
